# Golfers' Perspectives on Injury Prevention: A Qualitative Study on Factors Influencing Successful Implementation

**DOI:** 10.1155/tsm2/9501921

**Published:** 2025-07-23

**Authors:** Saskia Gladdines, Robert-Jan de Vos, Denise Eygendaal, Evert Verhagen

**Affiliations:** ^1^Department of Orthopaedics and Sports Medicine, Erasmus University Medical Center, Rotterdam, the Netherlands; ^2^Amsterdam Collaboration for Health and Safety in Sports, Department of Public and Occupational Health, Amsterdam Movement Sciences, Amsterdam University Medical Centers, VU University Medical Center, Amsterdam, the Netherlands

**Keywords:** golf, implementation, injury prevention, qualitative, warm-up

## Abstract

It is unclear so far how recreational golfers experience an injury prevention program in the real-life setting. A qualitative approach can be used to get insight into this implementation's complexity. The purpose of this study was to investigate the perception of recreational golfers on warm-up in general, their experiences with the Golf Injury Prevention Program (GRIPP), and their opinions on how to implement our injury prevention program in a recreational golf setting. We used an explorative qualitative design with a constructivist paradigm to underpin the study. Through convenience sampling, we invited 11 golfers assigned to awarm-up program in a golf-specific injury prevention trial. We conducted semistructured interviews following a standardized interview guide based on three predetermined topics: general warm-up, injury prevention, and implementation. Interviews were transcribed verbatim, and a thematic analysis was performed with ATLAS.ti software. Participants cited different reasons for engaging in a warm-up routine, including injury prevention, self-care, preparation, and performance optimization. However, conducting a warm-up was said to be influenced by the golf environment. The prevention program was deemed “feasible,” and the supplied materials were considered “sufficient.” Participants observed minor differences in the information channels available at golf clubs, which affect implementation. The role of the golf professional was recognized as crucial in promoting and facilitating the warm-up routine. Recreational golfers recognize the performance and health advantages of a warm-up routine, yet motivations for engaging in such activities vary. The social environment influences performance, and a golf professional can help implement this. These findings emphasize the importance of considering contextual factors when developing injury prevention programs in golf.

## 1. Introduction

Golf is a worldwide sport with a proven positive impact on physical health and mental wellbeing [1]. The obvious downside of playing sports is the risk of injuries. In golf, there is moderate injury risk when compared with other sports [1, 2]. Several studies among recreational golfers have reported an incidence for golf injuries up to 42.2% [3–9]. The injuries are most frequently located in the back and upper extremities in golf [1]. Warm-up exercises have been proven to be an effective measure of reducing injuries [10]. While the preventive effect of an intervention program has not yet been tested in golf, a warm-up program before playing golf could also potentially reduce injuries [11]. Almost 80% of golfers believe a warm-up will reduce injuries. Despite this, less than 1% knew what kind of warm-up they could perform before practice or play to prevent injuries [9]. Previous research suggested that golfers might be willing to perform a warm-up if they are shown how, as golf injury prevention programs were recently available [12]. A novel golf injury prevention program was developed just before the start of this qualitative study [13]. Most newly introduced injury prevention programs are vulnerable to the so-called “implementation gap.” This gap between research findings and practice implementation can be reduced by using the Knowledge Transfer Scheme (KTS) [14].

Therefore, the implementation context of a specific program needs careful consideration [15]. This can be done with, for example, the widely adopted Reach Efficacy Adoption Implementation Maintenance (RE-AIM) framework [16]. Key factors for successful implementation address end-users' behavior, beliefs, and attitudes [17, 18]. Getting insight into the perception of end-users about what is happening during the performance in the real-life setting (performance-as-done) compared with the developed performance protocol (performance-to-imagine) is essential [19]. Another approach is needed to understand this complexity in sports injury prevention research [20, 21]. Hence, a qualitative approach is often used in healthcare to understand patients' behaviors and beliefs or to get insight into complex processes [22, 23]. However, the use of qualitative research is limited in the evaluation of sports injury prevention programs [24]. There is no qualitative exploration of the vision, barriers, and facilitators of a warm-up program specifically for golfers.

This qualitative study explored and described the perception and perspectives of golfers about (1) warm-up in general, (2) their experiences and behavior with a golf-specific injury prevention program, and (3) the best way to implement an injury prevention program on a golf course.

## 2. Methods

### 2.1. Design

The current study is an explorative qualitative study to understand the factors influencing the implementation of a recently designed injury prevention program for golfers. A constructivist paradigm underpins the study. According to this paradigm, individuals actively construct knowledge and meaning in this paradigm through their experiences and social interactions [23]. We adhered to the recommendations for qualitative studies based on the Consolidated Criteria for Reporting Qualitative Research [25]. The medical ethical committee of Amsterdam University Medical Center (UMC) confirmed that the Medical Research Involving Human Subjects Act (WMO) does not apply to our study (W21_046 # 21.140, Addendum number 1).

### 2.2. Participants

Participants were recruited through convenience sampling from golfers participating in a study on a golf injury prevention program [13]. Golfers were assigned to the control group (perform warm-up as usual or no warm-up) or the intervention group (perform the injury prevention program). Only the golfers assigned to the intervention group were recruited for the current qualitative study around the injury prevention program. The injury prevention program is developed following the KTS method, focusing on preventing injuries to golfers' back and upper extremities. The effectiveness of the preventive intervention program in reducing injuries in recreational golfers is being assessed in a randomized controlled trial (RCT). Golfers assigned to the intervention group performed the developed injury prevention program during the 5-month follow-up period. The program consisted of six exercises, with 10 repetitions for both sides and a maximum duration of 8–10 min. It is an unsupervised program delivered by card and video instructions to the golfers [13]. The inclusion criteria of the golf injury prevention study were a World Handicap System (WHS) handicap of ≤ 36; ≥ 45 years of age; a playing frequency of at least nine holes a week; willingness to perform the injury prevention program; and understanding of the Dutch language [13]. In the final study questionnaire and the study newsletter, intervention group participants were invited to participate and share their experiences about the injury prevention program. If a potential participant gave notice of interest, an information letter on the study's background and goals was sent by email. The golfers were enrolled in the study in the order they responded positively to participate. After a positive response, digital informed consent was provided, and the interview was scheduled.

### 2.3. Reflexivity

SG is a female physical therapist, clinical health scientist, and an experienced golfer. EV is a male sports scientist and epidemiologist who is experienced in conducting qualitative research and has no experience playing golf. RJDV is a male sports physician experienced with treating a broad spectrum of sports injuries and a clinical researcher with no experience in playing golf. DE is a female orthopedic surgeon and sports consultant with limited experience in playing golf. This variety in the authors' backgrounds supports the neutrality of our findings.

### 2.4. Data Collection

Data were collected through individual semistructured interviews between December 2021 and March 2022. Participants were asked to participate based on the order of registration and their interest in participating. SG and EV developed the interview guide (Table 1). It covered three themes: (1) the general vision of warm-up about a warm-up for all sports, (2) preventive behavior and experiences with the golf injury prevention program, and (3) how to implement it in the participants' opinion. SG conducted all interviews and did not know the interviewees' participants. Two pilot interviews were performed to familiarize herself with the interview guide and interviewing techniques. These pilot interviews were not used for analysis. The interviews were performed in the Dutch language. Based on the participant's preferences, the interview was held via phone (*n* = 8) or online video conferencing using Zoom or Teams (*n* = 3). The participants' answers were summarized, repeated, or rephrased during the interviews to check if the interpretation was correct. All interviews were audio-recorded (Philips Voicetracer DVT6610) and transcribed verbatim after the interview. The interviews were stored for analysis at Erasmus Medical Centre.

### 2.5. Data Analysis

A thematic analysis was performed [26]. Researcher SG open-coded seven interviews using ATLAS.ti software (Scientific Software Development, Berlin, Germany; V.9). Based on the answers of the participants, specific topics were identified within the 3 predefined themes: (1) warm-up in general, (2) experience with the golf injury prevention program, and (3) implementation. During five meetings, EV critically reviewed the codes and categories and discussed them with SG until a consensus was reached. Subsequently, topics and subcodes were identified, discussed, and adapted if necessary. After consensus on the main codes, SG coded the remaining four interviews, which were used to verify whether saturation was reached. The quotes presented were translated from Dutch to English for this manuscript.

## 3. Results

### 3.1. Participant Characteristics/Demographics

All participants of the intervention group (*n* = 160) in the running RCT were invited to participate in this qualitative study. The first 11 responders (males: *n* = 5 and females: 6) were invited for an interview. The last 4 interviews were used to confirm that data saturation was reached. These 11 included recreational golf players had a mean age of 64.3 years (range: 53–71 years), a mean golf experience of 23.3 years (range: 2–44 years), and a mean handicap of 20.2 (range: 5.9–31.0). The mean duration of the interviews was 23.0 min (range: 17.4–30.0 min).

### 3.2. Summary of Results

**Figure 1:**
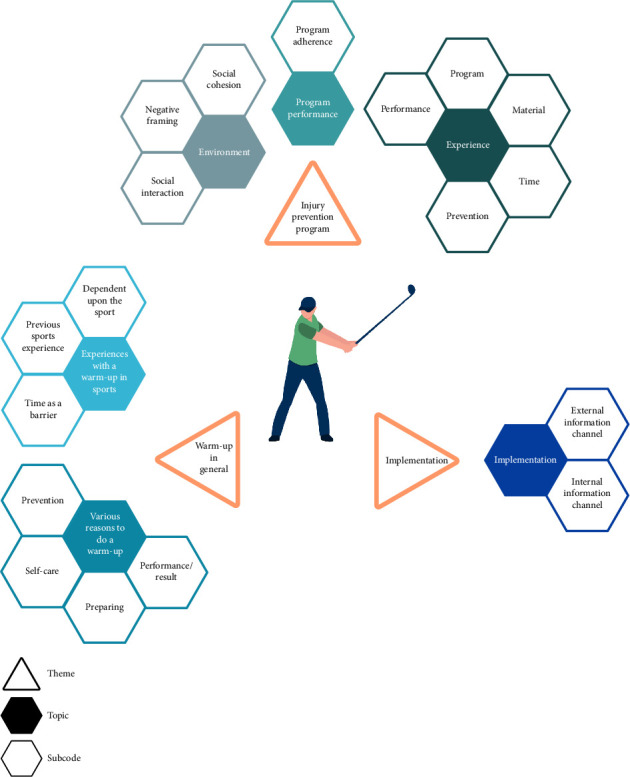
Themes, topics, and subcodes.

Our interview guide revolved around three main themes: warm-up in general, the injury prevention program, and implementation. The topics and subcodes derived for each theme are depicted in Figure 1 and are supported by exemplary interview quotes in the following (Tables 2, 3, and 4).

#### 3.2.1. Theme 1: Warm-Up in General

In this first theme, participants shared their experiences with and practices of warm-up before sports participation in general (Table 2). In this theme, we extracted the two main topics, “experiences with a warm-up in sports” and “various reasons to do a warm-up.”

Participants stated that, in their experience, warm-ups are sports-specific and sometimes already integrated into their training. Some participants also admitted to performing a warm-up before one sport but not for another without providing a further reason. The conduct of the warm-up was said to be positively influenced by the participants' sports experience. For some participants, the warm-up was simply a standard way to commence any sports activity. However, despite their standard warm-up conduct, participants mentioned time as a main barrier. Out of their experiences, they considered their time well invested but also noted that they had to “get used to it” to perform a warm-up in general. 

Participants mentioned various reasons for performing a warm-up in general. These included preventing injury and self-care, preparing themselves for exercise, and improving their performance. Not only was the prevention of injuries mentioned, but some participants saw performing a warm-up as taking care of themselves. None of the participants named these four reasons simultaneously, and each had their reason(s) for performing a warm-up in general before exercise.

#### 3.2.2. Theme 2: Experience With the Golf Injury Prevention Program

Participants described their experiences with the injury prevention program in this second theme (Table 3). In this theme, we extracted the three main topics: “social environment,” “experience,” and “program performance.”

The social environment is influencing the performance of the injury prevention program. Some participants experienced social interactions with other golfers when performing the injury prevention program. This interaction consisted of being watched, asking questions, and explaining the warm-up program to other golfers. Some participants performed the warming-up simultaneously with other golfers or recognized other golfers when performing the injury prevention program. It was also mentioned as a possible barrier because other golfers could watch the warm-up performance. Several participants could distinguish between the influence of social cohesion and related negative framing. One participant mentioned that she conducted the program in the locker room or at home to avoid such social interactions.

Participants mentioned their experience with the injury prevention program in several aspects. Most participants described the level of the program as easy to perform. Some participants mentioned that the program was accessible and addressed all aspects needed to play golf. The available material (video and instruction card) was comprehensive and supported the performance of the injury prevention program. Several participants mentioned it takes time to perform a warm-up, but that is part of the game when performing a warm-up. Some participants experienced a performance improvement, while others noted no difference. They mentioned that performing an injury prevention program influences golf performance with prepared muscles, which leads to better performance on the golf course. Some participants were golfers with current injuries, which did not prevent them from participating in golf. They noted that they experienced less pain when performing a warm-up before playing golf.

Some golfers just performed the program as received, and others adjusted the program due to physical limitations or pain. Mostly, they added some additional exercises to it.

#### 3.2.3. Theme 3: Implementation

In this third theme, participants described their opinions about implementing the injury prevention program for other golfers and golf clubs (Table 4). We extracted one main topic: “implementation.”

The participants mentioned golf.nl and the Dutch golf paper as communication options. These are external information channels from outside the golf club. Golf.nl is a Dutch Golf Federation channel with an online website, informative emails, and an online app to register the golf scorecard.

Participants stated that implementation options were directly related to the golf course or club. Those are listed as subcode: internal information channels. Golf clubs and courses have different channels to contact their members, such as board, commission, shop, club manager, newsletter, and website. Based on the answers of the participants who were members of different clubs, each club/course had a slightly different arrangement in the form of governance. However, in general, they all inform their members about relevant golf items using the internal channels of the golf club/course.

The participants believe an important internal information channel is the golf professional. Participants mentioned that the golf professional has much influence on a golfer. One participant mentioned that every golfer meets a golf professional at the start of their golfing career because every golfer requires lessons. As several participants mentioned, the golf professional sees many golfers.

## 4. Discussion

This qualitative study explored golfers' perceptions and experiences about a general warm-up, how an injury prevention program is received on the golf course, and how it can be further implemented in the future in the opinion of the end-users. Our findings provide insights into three main themes.

### 4.1. Relevance of the Golfer's Visions

Information about effectively implementing an injury prevention program within the golfer's context is important. Because of the complexity of implementing a program in the real world, the participants' perspectives on the delivery and setting in which it is performed have unique circumstances for each sport [27]. Different contextual factors influence the perspective of adopting measures and supporting maintenance [20]. The information gained in this study will give insight into the circumstances of the golfer (end-user).

### 4.2. Influence of Previous Warm-Up Experience

Participants mentioned various reasons for performing a warm-up and their experiences with a warm-up program depending upon the sport practiced and their previous sports experience. A previous experience with a prevention program in football positively improved the attitude of coaches about the warm-up [28]. Golf players are generally older and have likely previous experiences with multiple practiced sports during their lifetime [29]. Although golfers mentioned there is variety in performing a warm-up dependent upon the sport, the experience of performing in previous sports seems to normalize performing a warm-up. Reasons to perform a warm-up, in general, could be prevention, self-care, preparation, and performance. Comparing this with previous literature is challenging because general experience with a warm-up is barely described. However, previous research showed that the reasons for a golfer to perform a warm-up are to increase performance and prevent injuries [12]. While previous injury experience influences the attitude and perception of the warm-up. Previous injuries for adult runners and youth basketballers facilitated a deeper engagement with the warm-up [30, 31]. Golfers generally did not mention this as a reason to engage in a warm-up. However, it was mentioned as a reason to perform the injury prevention program.

### 4.3. The Environment Influences the Performance of the Program

The social environment was identified as a topic that influences behavior and the performance of the injury prevention program. Participants mentioned that being watched by other people could be a negative point. One participant mentioned performing the warm-up in the locker room for this reason. However, most participants mentioned being able to shut down. Previous literature describes how environmental context and behavior can influence preventive measures [17]. In team sports, there is the influence of different team members: coaches, captains, and subgroups of team members who have their perspectives on warm-up [30]. In golf, there are more sport-specific circumstances. There is interaction with a smaller and different audience, namely, flight members (groups of up to four golfers playing together) and or other golfers on the course. Most golfers gain information about warm-ups from other golfers [9]. From this viewpoint, there is a larger individual motivation part in golf, which is influenced by social interaction [18]. Experienced social cohesion was a facilitator to perform the warm-up program. It can also be a barrier when the interaction is negative. A golfer must have a clear opinion and persistence to shut down it. Golfers' behavior and the environment's influence are essential factors in the performance of the injury prevention program.

### 4.4. Time Is No Barrier

Another factor that may influence the adoption of the program is time. Participants mentioned that performing a warm-up takes time. In individual recreational runners, time was seen as a barrier, and a review of injury prevention studies showed time was seen as a barrier by coaches [18, 31]. Not having enough time was a reason 36% of the golfers did not perform warm-ups in golf [12]. For the injury prevention program, time seems to be not a barrier to performance. The program was built around the athlete, and warm-up sessions needed approximately 5–10 min, so the golfer could perform the warm-up while waiting on the tee-box for the tee time [13, 32]. It might be that this integration diminished time as a barrier. Another factor is that participants mentioned the program was easy to execute, it was accessible, had supporting material, and increased their performance. These elements are seen as facilitators in the performance of a prevention program and are recommended to increase implementation [18, 31, 33].

### 4.5. How to Implement the Injury Prevention Program?

The golfers mentioned internal (club) and external information (nationwide) channels to inform future program users. These channels can be used separately or simultaneously. The golf club is one of the internal information channels. The clubs all have a club board but slightly different management constructions and ways to approach their members. In addition, the setup of clubs is different, with or without a shop, reception, and caddie master. These locations are mentioned as good options to inform golfers and are, in all cases, locations with personal interaction. Another moment to interact with the golfers is during club activities or competitions. Nonpersonal interaction channels include the website, club magazine, posters, and social media. Another personal internal information channel at a golf club is the golf professional. The golf professional is someone with golf knowledge who teaches golfers the principles of golf and has, therefore, influenced them. Almost all mentioned golf professionals as essential factors in implementing the program. However, it is interesting that golfers noted that golf professionals have yet to tell them about a golf warm-up during their lessons. In team sports, the coach's role is essential to implement a program successfully [18]. The golf professional is a less frequent contact person for golfers but still has a role model function similar to a team sports coach. Golf professionals might need additional training to adequately inform golfers about the program.

**Figure 2:**
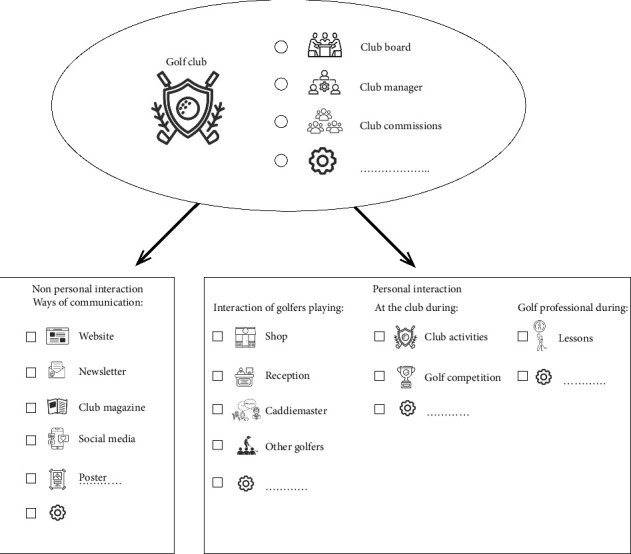
Potentially available information channels at golf clubs to inform about the injury prevention program.

Since each club has its unique structure, it might be important to plan out the structure needed for each club to successfully implement an injury prevention program at a particular golf club. In Figure 2, an overview is provided, including a figure of all the available channels for golf clubs mentioned by the golfers. The external information channel has a much broader reach with multiple options to inform the golfers regularly and repeatedly. The Dutch golf paper and the Dutch Golf Federation are options for golfers to be informed with golf.nl. Golf.nl has a reach with its website, app, and newsletter. For example, a golfer must use the app to enter a scorecard. This likely enhances the uptake of an injury prevention program.

## 5. Strengths and Limitations

A strength of this study was the multidisciplinary team, which had wide experience in injury prevention programs research and was involved in the research to provide a broad perspective. We strictly adhered to the existing recommendations for qualitative studies. The interviewer's (SG) personal experience with golf created a strong rapport with the golfers during the interviews. This may have contributed to more connection with the golfers and in-depth answers in golf-related terms. A potential disadvantage could be that the three themes were predefined, and one researcher (SG) influenced the study's outcome too much. Therefore, the analysis was discussed and validated during multiple meetings and discussions. The other authors had no or little golf experience but were extensively involved in sports medicine research. Therefore, the findings were neutralized by the other authors and enhanced conformability.

We should know that the assessed injury prevention program was previously tested on correct performance. Also, the end-users were involved during the development process [13]. It could be that those factors were of influence. The end-users in the development were recreational golfers, so caution is needed to use our program outside this setting. Professional golfers are having higher incidence and prevalence rates than amateur golfers [34–36]. We chose to include golfers with a handicap of 36 and lower, as golfers above this handicap are typically seen as beginners [37]. They will have their own injury risk that differs from more experienced players. It is suggested that different injury patterns are reason for this, such as playing frequency and biomechanics [35, 38]. The influence of suboptimal swings with biomechanical issues was out of the scope of the RCT. We previously mentioned in the RCT protocol that all golfers are amateur golfers of 45 years and older; they each likely have their own personal body limitations and injury history, contributing to their unique swings [13].

The average handicap in this study is similar to the handicap in the RCT. The population included in this study could be seen as a representative sample of club golfers in the Netherlands. In this study, we approached golf club members with a home course. In the Netherlands, 52% of the golfers are members of a golf club with a home course [29]. Therefore, we need to take caution in interpreting the results for the group of golfers who are members of a golf club without a home course, also called virtual clubs. Those golfers pay a green fee to play on the course and have a lower playing frequency [39]. Our results might not be extrapolated to the group with a virtual home course. Also, outside the Netherlands, the results should be interpreted cautiously. There might be a different structure and setup of golf clubs and contact between golfers in other countries. It is plausible that the group with a home course is also abroad, playing with a higher frequency. Therefore, specific elements in the club structure in our study are identifiable and extractable from a golf club abroad.

## 6. Practical Implications

The outcomes of our study provide an understanding of golfers' perception of influencing factors around implementing a recently designed injury prevention. When implementing a program for a group of older athletes, it is necessary to pay attention to their different experiences and perceptions during their lifetime in sports with a warm-up. It is shaping their attitudes toward warm-ups in golf. The motivations for a golfer to perform a warming-up are self-care, performance enhancement, preparation, and injury prevention. But previous experiences can influence their perception and behavior with a warm-up and need to be considered during program development.

The social environment can be an essential facilitator, in the behavior and adherence to the program. The performance of an unsupervised program has some different challenges. Social cohesion and social interaction could be seen as facilitators while negative framing could be considered as a barrier. Another facilitator could be implementing this new intervention, the participants saw this program as feasible, and supplied with sufficient material. Also, it is extraordinary that the golfers did not see time as a barrier, a common problem in previous research [18, 31]. This might be because the designed warm-up is brief (5–10 min) and can be integrated into their routine while waiting on the tee-box.

Future golf injury prevention programs need to employ the possible supportive role of a coach/trainer. Although our program is unsupervised, golfers mentioned that golf professionals are an important information channel for implementing the program. The golf professional is part of the internal information channel (club). It is crucial to reach the potential users by getting insight into all internal (club) and external (nationwide) information channels to disseminate a program to and encourage participation, especially because there are slight differences in the governance structures of the various clubs/courses. It is necessary to get a quick insight into the club structures and information channels to use the full potential of information channels.

## Figures and Tables

**Table 1:** Interview guidelines with the three main themes and related questions.

**Warm-up in general**

What is the benefit for you of performing a warming-up program?
What is, in your opinion, positive about performing a warm-up program?
What is, in your opinion, negative about performing a warm-up program?

**The injury prevention program**

What do you think of the injury prevention program?
If you modified the program, what kind of modifications did you make?
Why did you make these modifications?
What preparations have you made to perform the injury prevention program?
What do you think about the amount of time you spend on the injury prevention program?
What do you think about the difficulty level of the program?
What are the strong points of the injury prevention program?
What are the weak points of the injury prevention program?
What is the influence of the injury prevention program on your golf performance?
What are the reasons for performing injury prevention before playing golf?
What are the reasons not to perform injury prevention before playing golf?
In which locations are you performing the exercises and why?
What motivates you to use or not use the injury prevention program in the future?

**Implementation**

Who can inform golfers unfamiliar with the injury prevention program on your golf course about the program?
Why can they inform the golfer about the injury prevention program?

**Table 2:** Topics, subcodes, and exemplary quotes on the main theme “warm-up in general.”

Topic	Subcode	Exemplary quote
Experiences with a warm-up in sports	Dependent upon the sport	Golfer 3: “For example, when it comes to playing golf, I do a warm-up, and for cycling, I also do specific exercises. But for running, you would not do that”
		Golfer 10: “When I played tennis, I did not do any warm-up. Maybe I did one during the training sessions, where they might pay some attention to it. But when I played a casual game of tennis, I usually did not. And besides, I have mostly done individual sports like fitness, and I do pilates, you know, that kind of thing. But yeah, warm-up routines are usually integrated into the program”
	Previous sports experience	Golfer 2: “I have always been very active with playing sports, so it is a routine for me to do some exercises to stretch my muscles”
		Golfer 5: “I have practiced other sports in the past, and it was always like…if you want to do it well, you just need to be flexible, that is it”
	Time as a barrier	Golfer 1: “You need to ensure you are on the course on time to warm up a bit before you start”
		Golfer 5: “I have been involved in various sports for a long time. So, I have become accustomed to it. But I know I found it difficult to make time for it every time, especially in the beginning”
		Golfer 4: “Well, the only negative point for people in practice is that it takes time, but that is time well invested, and I think it is essential to communicate that aspect too”

Various reasons to do a warm-up	Prevention	Golfer 2: “The most important thing for me is preventing injuries and pain complaints”
		Golfer 6: “In general, I think it is about preventing injuries and overuse …well…primarily preventing injuries”
	Self-care	Golfer 7: “Well, I take good care of myself”
	Preparing	Golfer 6: “Preparing your body for an effort”
		Golfer 1: “Getting some movement before you can use your body as optimally as possible is my experience”
	Performance/result	Golfer 5: “Getting a good warm-up in sports leads to optimizing your sports performance, which is my experience”
		Golfer 1: “You cannot perform well right away from a very passive starting position; that does not work”

**Table 3:** Topics, subcodes, and exemplary quotes on the main theme “experience with an injury prevention program.”

Topic	Subcode	Exemplary quote
Social environment (behavior)	Negative framing	Golfer 8: “The next negative point to consider is that when you are at the tee, you start doing all sorts of exercises, and I notice that many people passing by and look at you as if to say, “What on earth are you doing?” I do not pay any attention to it, but I can imagine that other people might think, “I had better do that somewhere else or not at all.” That is the only negative thing I can think of”
	Social interaction	Golfer 10: “There were, of course, people who wondered what those ladies were doing, waving those clubs around. So, we did explain it to them”
		Golfer 11: “But I think you must overcome a certain self-consciousness to step up and do those exercises there. At some point, I transitioned from…well…either to the locker room or simply at home before I left…otherwise, I would not do it”
	Social cohesion	Golfer 6: “Meanwhile, when more people do it, you also recognize each other, and then you talk about it”
		Golfer 5: “So, there were four of us on the Sunday flight, just doing that warm-up beforehand. So, I found it quite positive that everyone was starting to do that, and the acceptance was much higher”
		Golfer 9: “I was on another golf course and saw a few women (laughing tone) doing those exercises, and I thought, hey, that is funny. I do those, too”

Experience	Program	Golfer 3: “The accessibility of that program”
		Golfer 6: “What I find positive about it is that you can do it quickly. So, those 5 min do not take much time, and you notice that you are already warmed up quite quickly and with the right muscles…well…everything kind of falls into place”
		Golfer 2: “Well, the exercises incorporate many movements from the muscles you use during golf”
	Material	Golfer 7: “Well, it has helped me tremendously; I appreciate it. The exercises are very good. There were good instructions, which helps,…so that is what I liked the most”
		Golfer 6: “I initially watched the video once. Then, I started doing it based on the card instructions. After that, I rewatched a part of the video because I did not fully understand one of the exercises. That is how I did it”
		Golfer 1: “I watched the instructional video, put that card in my bag, and it is still in my bag, but I do not need it anymore because I know the exercises by heart now”
		Golfer 2: “The list supports you in doing the exercises”
	Time	Golfer 3: “Well, you could say it takes time, but I do not consider that a weakness because it is just how it is”
	Performance	Golfer 8: “My experience is that it helps to be warmed up for the first holes already”
		Golfer 5: “The result that I hit (the ball) further”
		Golfer 2: “My swing is improving, and I can play better through the course”
		Golfer 1: “Occasionally, of course, it does happen that I start without doing the exercises. Then I do not notice much difference, to be honest”
	Prevention	Golfer 7: “Well, I have less pain and take good care of my body when I play. Because golf is, of course, a peculiar sport in terms of movement, and I am a bit older, so yeah, I think it's important that I exercise the muscles that are needed because I notice that if I do not do it, I have a lot of pain afterwards”
		Golfer 10: “I do not know if it has saved me from injuries or anything, but at least every time I think…well… it is still good to do it, and I keep doing it”
		Golfer 2: But when I do those exercises, I…uh…have the lowest risk of getting complaints along the way or ending up with back problems at home”

Program performance	Program adherence	Golfer 8: “I always faithfully followed the program on that instruction card”
		Golfer 9: “Well, not adjustments, but yes, I have made a few adjustments, like adding some of my exercises, like the leg swing”
		Golfer 6: I might do additional exercises if I have more time

**Table 4:** Topics, subcodes, and exemplary quotes on the main theme “implementation.”

Topic	Subcode	Exemplary quote
Implementation	External information channel	Golfer 8: “Golf.nl seems to be another one, and as golfers, we often use it to enter our scores and receive emails from there”
		Gofer 9: “The Dutch Golf Newspaper is also often available at the club”
	Internal information channel	Golfer 10: “But of course, you want to reach the entire target audience, all the members. In that case, you just need to approach the board, use the website, or ask for attention in the newsletter. We have a monthly newsletter at the club, for example”
		Golfer 3: “It starts with the golf pro. I think that is what it is. Yeah, they have a lot of influence on everyone”
		Golfer 5: “Well, they are the ones who give lessons, and it seems that they are the ones who ensure that someone can hit well and all those things like accuracy and distance. They should also include this aspect to ensure people enjoy their golf game. That is why I say a pro”

## Data Availability

The data that support the findings of this study are available on request from the corresponding author. The data are not publicly available due to privacy or ethical restrictions.
